# Evaluation of Screened Lignin-degrading Fungi for the Biological Pretreatment of Corn Stover

**DOI:** 10.1038/s41598-018-23626-6

**Published:** 2018-03-29

**Authors:** Yingjie Su, Xiaoxiao Yu, Yang Sun, Gang Wang, Huan Chen, Guang Chen

**Affiliations:** College of Life Sciences, Jilin Agricultural University, Changchun, 130118 P. R. China

## Abstract

The biological pretreatment of lignocellulosic biomass is a low-cost and eco-friendly method for facilitating enzymatic hydrolysis. In this study, strains with lignin depletion capability were screened using a high-throughput screening method. Sixty-three strains were screened out and *Myrothecium verrucaria* secreted three lignin-degrading enzymes simultaneously during the bio-pretreatment process. The activity levels of laccase, lignin peroxidase and manganese peroxidase were 6.61, 0.78 and 1.31 U g^−1^ dry biomass. The content of lignin in corn stover decreased by 42.30% after bio-pretreatment, and the conversion rate increased by 123.84% during the subsequent saccharification process in comparison with the untreated corn stover. Furthermore, the effects of bio-pretreatment on the structure of corn stover were presented using a scanning electron microscope (SEM), Brunauer-Emmet-Teller (BET), X-ray diffractometer (XRD) and Fourier transform infrared spectroscopy (FTIR). The results showed that *M*.*V*. is a promising lignin-degrading fungus. This research demonstrated an efficient pretreatment approach for enhancing the enzymatic saccharification of corn stover.

## Introduction

Chemical energy shortages and increasingly severe environmental pollution have forced people to explore renewable green energy^1^. Lignocellulosic materials, which consist of cellulose, hemicellulose, and lignin, are suitable candidates because they are carbon-neutral, abundant, and affordable^2^. The biorefinery technology based on the sugar platform is an effective way to utilize the raw materials of lignocellulose. Corn stover, rice straw, and other lignocellulosic feedstock are usually converted into monosaccharides, which are in turn converted into biobased products by microbial fermentation^3,4^. Enzymatic hydrolysis can be used to convert the cellulose in corn stover into monosaccharides^5^. However, cellulose is hard to digest due to the covalent interactions and linkages between lignin and hemicelluloses. Lignin is a kind of high complex aromatic polymer with a three-dimensional structure formed by a benzene-propane unit through the ether bond and the carbon-carbon bond. It is found in all vascular plants including herbaceous species, which provides rigidity, support, and protection to the plants^6^. As a macromolecule and highly branched polymer, lignin forms the lignin sheath and provides a physical barrier and is recognized refractory substance that is resistant to enzymatic hydrolysis^7,8^. In addition, lignin could physically block cellulase, preventing it from being combined with the substrate in a process known as non-productive binding^9^. Therefore, though the depletion of hemicellulose would also enhance the conversion rate of cellulose in biomass, the depletion of lignin was a crucial method for obtaining fermentable sugars from the lignocellulosic biomass^10^.

Pretreatment is essential for the reduction of biomass recalcitrance and functions by destroying the lignin structure and then increasing the enzyme accessibility of cellulose^11^. Delignification can occur through chemical, physical and biological methods. The lignin contents can be reduced by approximately 50% by physical and chemical pretreatment, but both the physical and chemical methods have disadvantages in terms of their high cost and tendency to cause secondary pollution. The biological method, with the advantages of simple operation, low cost and environmental friendliness, is considered a promising pretreatment method^12,13^.

Many species of microorganisms can degrade lignin in nature. The complete degradation of lignin is considered to result from the interaction of certain fungi and bacteria, in which fungi play a major role. White rot fungi, brown rot fungi and soft rot fungi can degrade lignin, and white rot fungi can effectively mineralize lignin, according to the reports^14^. Some white rot fungi can simultaneously degrade lignin and polysaccharides, resulting in the loss of carbohydrates, while other white rot fungi can selectively degrade lignin^15^. It is, therefore, necessary to choose suitable strains to identify the most potent strain for ideal pretreatment. The optimal strain should maximize lignin removal and minimize polysaccharide hydrolysis in corn stover^16^.

The degradation of lignin mainly depends on the action of related lignin-degrading enzymes, such as laccase (Lac), lignin peroxidase (LiP) and manganese peroxidase (MnP). These three enzymes act synergistically to degrade lignin in the biomass. LiP and MnP degradations of lignin are dependent on H_2_O_2_ production by H_2_O_2_ enzymes during lignin degradation, while Lac can catalyse the phenolic dimer methoxy-substituted quinone, which is the O_2_ source required for H_2_O_2_ production. Lignin is degraded by the interaction of three lignin-degrading enzymes^17^. According to the available reports, Lac occupies an important place in the lignin degradation process^18^.

In this research, corn stover was selected as the research material. Strains with degradation properties for lignin were screened from nature. The potential to improve the ability of lignin depletion and enhance the saccharification of cellulose of the screened strain during the pretreatment process was investigated. The effect of the screened strain on the structure and composition of corn stover during the process of pretreatment were studied. An efficient strategy for enhancing enzymatic saccharification was established.

## Results and Discussion

### Determination of the alkali lignin removal rate of lignin degradation strains

Reported research on the screening of lignin-degrading strains has mostly focused on qualitative screening plates. Only one or two ligninases could be detected by this method, which cannot reflect the true performance of the lignin-degrading strains. The reaction of qualitative screening plates can only reflect the ligninase production capacity of the strain, while the size of the coloured circle and the enzyme activity are not necessarily positively correlated. Therefore, a quantitative lignin degradation test needed to be performed to assess the lignin degradation that occurred via the fermentation of the strain. Alkali lignin is a kind of lignin extracted from plant tissues with alkali, which is used to evaluate the capability of lignin degradation of fungi in this research. Barapatre studied the biodegradation of alkali lignin by *Aspergillus flavus* and *Emericella nidulans* and found that approximately 14.4% to 21% of alkali lignin reduction occurred in different mediums^19^. Kirk suggested that *Phanerochaete chrysosporium* (*P*.*C*.) efficiently degrades the synthetic lignin in C-sufficient medium, and 22% of lignin decomposed into the total ^14^C of the ^14^CO_2_^20^. Therefore, it is feasible to evaluate the lignin degradation ability of the strains by alkali lignin removal rate.

**Figure 1:**
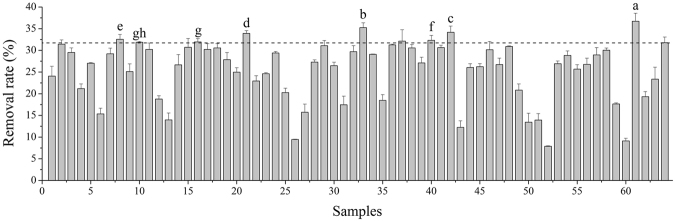
Alkali lignin removal rate (%) of mediums after pretreatment with 64 strains for 96 h at 29 °C. The data presented is average ± standard deviation for triplicate experiments for each fungal strain.

The isolated fungi were cultured in medium containing alkali lignin as the sole carbon source. The ability of the strain to utilize alkali lignin was determined. A total of 63 strains that could grow well in the alkali lignin medium were isolated and found to have alkali lignin removal ability. *P*.*C*. (the classic strain for lignin degradation) was used as the control group (No. 64). Removal rates of screened fungi for alkali lignin are shown in Figure 1. The removal rate of *P*.*C*. was 31.76%, and 8 strains showed a stronger ability for alkali lignin utilization: No. 8–32.57%, No. 10–31.88%, No. 16–31.94%, No. 21–33.92%, No. 33–35.24%, No. 40–32.32%, No. 42–34.18%, and No. 61–36.73%, the significance analysis of these data were also shown in Figure 1. Strain No. 61 showed a highest removal rate of up to 36.73%, which suggested that it had the strongest ability to utilize alkali lignin.

### Enzymatic saccharification of bio-pretreated lignocellulose biomass

**Figure 2:**
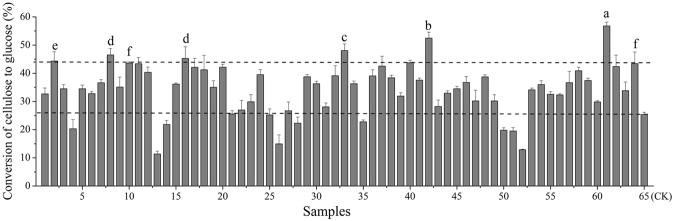
Conversion of cellulose to glucose after the enzymatic hydrolysis of bio-pretreated corn stover by 64 fungi. The data presented is average ± standard deviation for triplicate experiments for each fungal strain.

To determine the enzymatic hydrolysis and saccharification effects of corn stover bio*-*pretreated by the 63 screened strains and *P*.*C*., the conversion rate of cellulose to glucose was determined after 72 h enzymatic hydrolysis with cellulase and β-glucosidase. The conversion rate of cellulose in corn stover after bio-pretreatment was compared with that of untreated corn stover. The results are shown in Figure 2. We noted that 52 strains grouped into the enhanced group compared to the control, which may be because some of the lignin was modified or broken by bio-pretreatment, resulting in a significant increase in the susceptibility of the corn stover to enzymatic hydrolysis. Among them, the enzymatic hydrolysis of 7 strains was better than that of *P*.*C*., and the conversion rate was the highest at 56.81% following the pretreatment of strain No. 61, which was increased by 123.84% compared to the untreated corn stover. The same method to screen strains of enhancing the efficiency of enzymatic hydrolysis was used by Zhou, and *Phlebia sp*. BRFM 1123 was found to be particularly effective at increasing the release of fermentable sugar from miscanthus, with a maximum improvement of up to 62% of glucose^21^. The data provided by Dien also showed that the sugar recovery was negatively correlated with the lignin content^22^. This finding agreed with our results showing that the strains with a robust alkali lignin utilization capacity also had a higher sugar recovery rate. A nearly equal content of glucose was found in the 2 samples compared to the control, suggesting that there was no effect from the bio-pretreatment of these two strains. 9 strains grouped into the detrimental-effect group, perhaps because some of the cellulose in the corn stover was consumed by the bio-pretreatment process. It is also possible that some of the metabolites secreted during the bio-pretreatment process that inhibit enzymolysis were not eliminated well. This could feasibly reduce the efficiency of cellulose hydrolysis.

### Lignin-degrading enzyme production during the bio-pretreatment of corn stover

The activity of lignin-degrading enzymes was closely related to the degree of lignin degradation in corn stover because the enzymes secreted by strains broke down the heterogeneous compounds into small constitutive components. These components were consumed for the growth and metabolism of strains when cultivated in corn stover.

**Figure 3:**
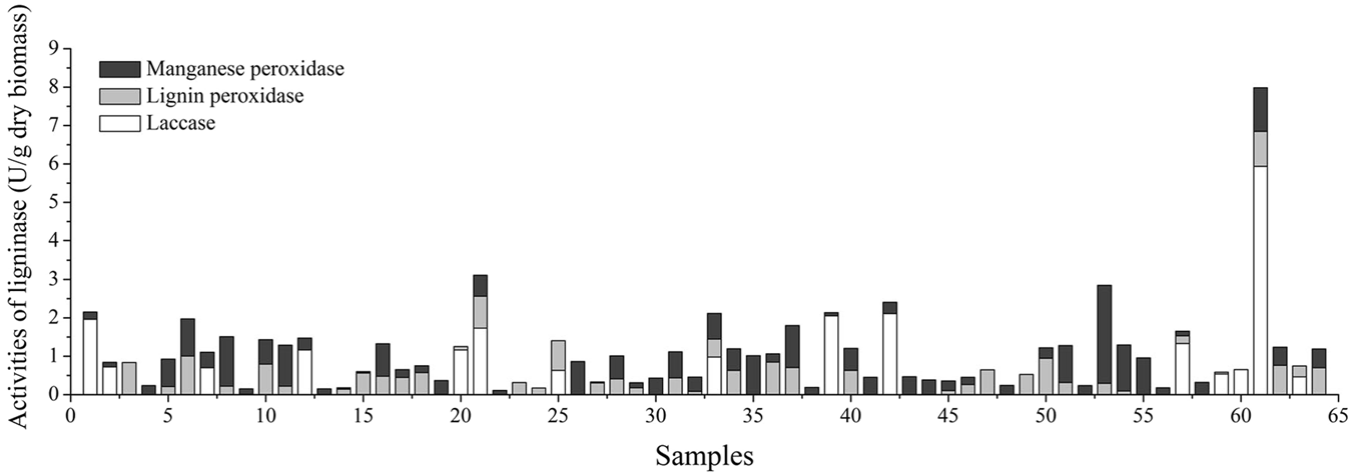
Ligninase activities of 64 strains after 14 days of solid-state fermentation on corn stover. The data presented is average ± standard deviation for triplicate experiments for each fungal strain. No. 1 to No. 63 stand for screened strains and No. 64 stands for the classic strain.

To obtain insights into the lignin-degrading enzymes secreted during bio-pretreatment, the activities of Lac, LiP and MnP were determined, as shown in Figure 3. In general, only one or two kinds of ligninase could be identified in most strains. The highest production of Lac was found in No. 61 and reached 5.93 U g^−1^ dry biomass. The highest production of LiP was found in No. 50 (1.09 U g^−1^ dry biomass), and the highest production of MnP was found in No. 53 (2.56 U g^−1^ dry biomass). The three enzymes were secreted simultaneously in strains No. 21, 33, 57 and 61. Higher levels of the three enzyme activities were measured in strain No. 61, which had higher lignin removal capacity and a higher conversion rate of cellulose to glucose in previous results. Though not all the lignin-degrading fungi could produce all of three kinds of enzymes, only one or two of them also had a certain capability of lignin degradation, No. 61 with high lignin depletion capability in our research showed higher three enzyme activities. Liu proved that the strain with the highest lignin degradation capability played a crucial role in bio-pretreatment^23^. Thus, strain No. 61 is a potentially promising candidate for the bio-pretreatment of corn stover. Only LiP and MnP were detected during the pretreatment process of *P*.*C*., which is consistent with the results of Zhang^24^. Kapich also proved that the wild-type strain *P*.*C*. ME-446 is able to effectively produce MnP and LiP in the lignocellulosic medium^25^.

### Strain identification

**Figure 4:**
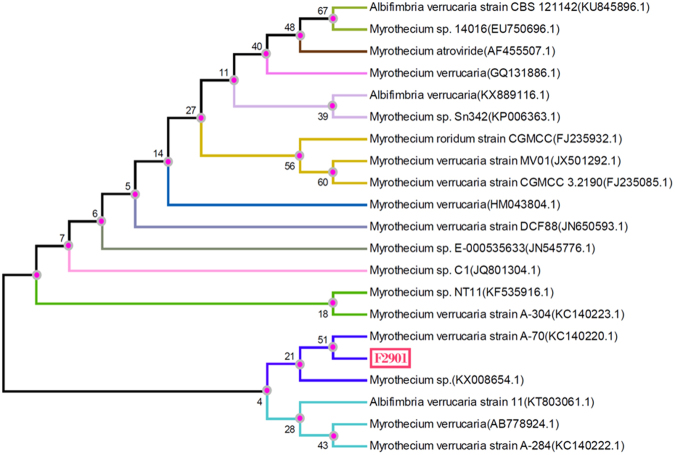
Phylogenetic tree analysis of *M*.*V*.

Molecular identification techniques were used to determine the species of strain No. 61. According to the ITS sequences reported in GenBank, the fungi had a high sequence homology with *Myrothecium*. The phylogenetic tree was constructed with ClustalX1.83 and MEGA4, as shown in Figure 4. F2901 was the experimental strain, and phylogenetic analysis was carried out after comparison with evolutionary species. The results showed that the experimental strain had the closest genetic relationship with *Myrothecium verrucaria* (*M*.*V*.), both of which were on the same evolutionary branch. The strain was identified as *M*.*V*. It can be seen from Fig. S1 that *M*.*V*. began the logarithmic growth period at 36 h and reached the stable period at 96 h.

### Lignocellulosic degrading enzyme production during *M*.*V*. cultivation

**Figure 5:**
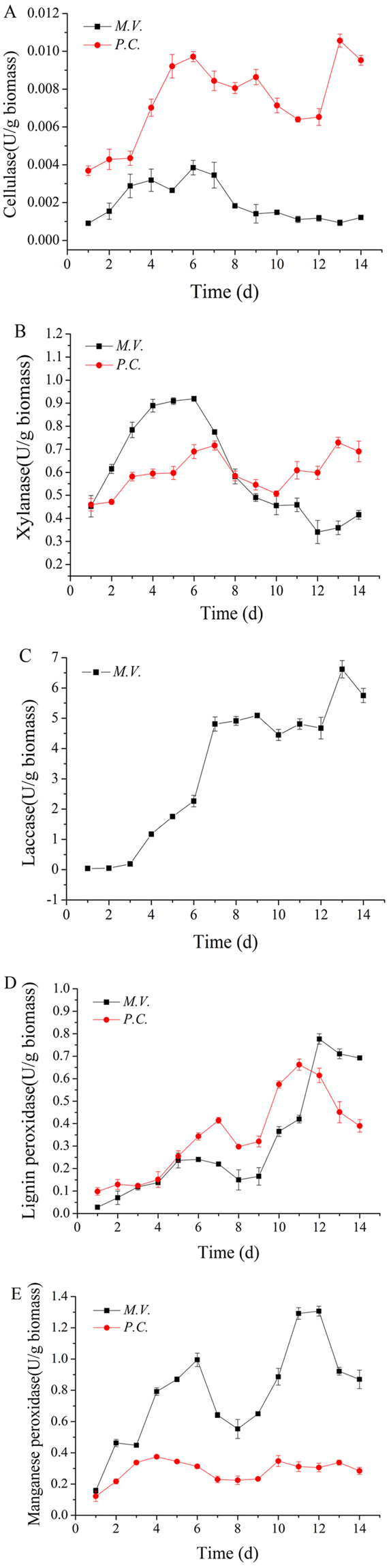
Lignocellulase activity of *M*.*V*. and *P*.*C*. (**A**) Stands for cellulase activity; (**B**) stands for Xylanase activity; (**C**) stands for Laccase activity; (**D**) stands for Lignin peroxidase activity; (**E**) stands for Manganese peroxidase activity. Each value is the mean of three replicates.

To understand the degradation of corn stover during the pretreatment of *M*.*V*., the enzymatic process of five enzymes (Cellulase, Hemicellulase, Lac, LiP, and MnP) related to the degradation of lignocellulose during pretreatment was determined and compared with that of *P*.*C*. The results are shown in Figure 5. It should be noted that little cellulase or xylanase activities was detected in the extract of *M*.*V*. fermentation broth. The cellulase activity of *M*.*V*. was 0.0038 U g^−1^ dry biomass at 6 d, which was too low to degrade cellulose. Therefore, the cellulose in the corn stover could be well preserved during the bio-pretreatment process. The cellulose activity of *P*.*C*. was a little higher, which was 0.0106 U g^−1^ dry biomass at 13 d. While the xylanase activity was higher, the peak activity appeared at approximately 6 d for both *M*.*V*. and *P*.*C*., reached 0.92 and 0.72 U g^−1^ dry biomass. The xylanase activity of *M*.*V*. was higher than that of *P*.*C*. Panaqiotou reported that the xylanase activity of *Fusarium oxysporum* reached 1216 U g^−1^ in the culture of corn stover^26^. The lower levels of cellulase and xylanase indicated the lower capability of degrading cellulose and hemicellulose. In contrast to *M*.*V*., the brown-rot fungi could not only modify lignin to a certain extent but also degrade the cellulose and hemicellulose of wood^27^.

Three enzymes associated with lignin degradation were simultaneously secreted by *M*.*V*., and high Lac activity could be detected. After fermentation for 13 d, Lac reached its maximum activity of 6.61 U g^−1^ dry biomass. Lac was not detected in the fermentation broth of *P*.*C*., which was consistent with the results of a previous study showing that *P*.*C*. only expressed LiP and MnP^28^. It has been reported that Lac played a positive role in lignin degradation. Liu proved that after a 36 d cultivation period by fungus *Pycnoporus sp*. SYBC-L3, nearly a 30% reduction in lignin content was obtained without a significant loss of cellulose and hemicellulose. And a considerable amount of Lac, as high as 6.3 U g^−1^, was produced^29^. The LiP and MnP activity of *M*.*V*. were higher than those of *P*.*C*., and the maximum LiP activity of *M*.*V*. reached 0.78 U g^−1^ dry biomass at 12 d, which was 18.2% higher than that of *P*.*C*. The maximum MnP activity of *M*.*V*. was up to 1.30 U g^−1^ dry biomass, approximately 3.7-fold higher than *P*.*C*., indicating that *M*.*V*. had a stronger ability to degrade lignin than *P*.*C*., perhaps because the enzyme was closely related to the degradation process. The similar delignification of corn stover by the white rot fungus *Ceriporiopsis subvermispora* in solid-state cultivation was researched by Wan, who confirmed that MnP and Lac were detected during the degradation of corn stover, while no LiP was detected during the corn stover decay process. MnP reached its maximum activity of 2.2 IU g^−1^ solid at 7 d. Its activity then decreased to approximately 0.3 IU g^−1^ solid after 28 days^30^. Indeed, the MnP activity was originally reported to be 0.2 IU g^−1^ and 0.1 IU g^−1^ in sugar cane and wood, respectively, by *C*. *subvermispora*^15^. Lignin in corn stover was degraded by the synergistic effects of three lignin-degrading enzymes. Therefore, the availability of cellulase and cellulose was increased and more sugars could be produced in the subsequent hydrolysis by cellulase.

### Composition analysis of lignocelluloses and phenols

The biomass loss of the corn stover was measured after bio-pretreatment and results were shown in Table 1. After a 14 d cultivation period by *M*.*V*. and *P*.*C*., the biomass losses were 23.67% and 20.35%, respectively. Liu evaluated 25 fungal strains for pretreatment of switchgrass and 5.08% to 31.34% biomass losses were observed^23^. These data indicated that some parts of corn stover were consumed by bio-pretreatment. While biomass loss could not directly represent the effects of the pretreatment, for the ideal pretreatment for enzymatic saccharification should be to minimize the content of lignin and to maximize the retention of cellulose.

**Table 1:** Effects of bio-pretreatment on major components of corn stover after fermentation for 14 d.

Sample	Biomass Loss (%)	Cellulose (%)	Hemicellulose (%)	Lignin (%)	Phenols (mg/L)
untreated	—	38.34 ± 1.05	18.95 ± 0.67	22.57 ± 2.13	134.57 ± 6.71
*M*.*V*. pretreatment	23.67 ± 0.52	48.79 ± 0.38	24.60 ± 2.79	16.99 ± 0.49	57.93 ± 1.56
*P*.*C*. pretreatment	20.35 ± 1.22	39.94 ± 0.66	21.92 ± 0.43	19.83 ± 0.21	120.03 ± 4.19

Notes: Values of cellulose, hemicellulose and lignin after bio-pretreatment were presented as the relative content. Degradation rate of cellulose after *M*.*V*. pretreatment = [10 × 38.34% − (10 − 10 × 23.67%) × 48.79%]/(10 × 38.34%) × 100% = 2.78%, the other degradation rate of each components after different pretreatment were calculated by the same method, and the degradation rate of cellulose after *P*.*C*. pretreatment was 17.02%; the degradation of lignin degradation rate after *M*.*V*. pretreatment was 42.30%; the degradation of lignin degradation rate after *P*.*C*. pretreatment was 30.04%. Each value was the mean of the three replicates.

To further understand the effects of bio-pretreatment on the compositions of corn stover, the quantity of cellulose, hemicellulose and lignin was determined using the NREL method, as shown in Table 1. The relative content of cellulose and hemicellulose increased from 38.34% and 18.95% to 48.79% and 39.94% and to 24.60% and 21.92% after pretreatment with *M*.*V*. and *P*.*C*., respectively, which agreed with the results of Mohanram, who had previously found that the amount of cellulose in rice straw was increased after the process of biological delignification by *Trametes hirsute* and *Myrothecium roridum*^31^. The relative content of lignin decreased from 22.57% to 16.99% and 19.83% after pretreatment with the two strains, indicating that both *M*.*V*. and *P*.*C*. have the ability to degrade lignin in corn stover. A 42.30% reduction was observed in the lignin content for *M*.*V*. pretreatment, and no significant loss in the cellulose and hemicellulose content. While for *P*.*C*. pretreatment, a 30.04% reduction in the lignin content and a 17.02% loss in the cellulose content were observed. A similar result of lignin depletion (34.1%) was determined by the pretreatment of *D*. *squalens*^32^. Previous studies have shown that the total amount of lignin degradation could vary between 12% (after 16 days of wheat straw fermentation) and 42% (after 35 days of cultivation in glucose-enriched wheat straw medium)^33,28^. Compared with these data, *M*.*V*. pretreatment in our study showed a higher delignification rate for corn stover, and the effect of lignin depletion was better than that of *P*.*C*. Meanwhile, cellulose and hemicellulose were conserved by the process of SSF, which could be converted to glucose in the subsequence procedure, indicating that *M*.*V*. fermentation was an ideal pretreatment method for lignin depletion and the efficient utilization of cellulose in corn stover.

Lignin is decomposed into phenol and non-phenol substances by lignin-degrading enzymes (Lac, LiP, and MnP). Phenolic substances can easily be degraded by Lac, and non-phenolic metabolites can also be degraded by Lac with the help of a suitable mediator. It has been reported that phenolic compounds can inhibit cellulase and other hydrolases^34^. Therefore, any phenols introduced with pretreatment must be removed to maximize the enzyme activity. The total phenol concentration in the corn stover before and after pretreatment was measured using the method described by Folin-Ciocalteu. The results are shown in Table 1. The total phenolic concentration decreased by 56.95% and 10.80% after pretreatment with *M*.*V*. and *P*.*C*., respectively. Jurado and Moreno demonstrated that phenolic compounds were greatly reduced or eliminated during Lac treatment before enzymatic hydrolysis^35,36^. Oliva-Taravilla studied the effects of Lac treatment on steam-exploded enzymatic hydrolysis and proved that the total phenolic content decreased by 80% after Lac treatment^37^. Pretreatment of *M*.*V*. removed 46.15% more phenols than *P*.*C*. This might be because *M*.*V*. secreted more Lac than *P*.*C*.

### Microstructure analysis

The physical surface structure of untreated and bio-pretreated corn stover was studied by SEM micorgraphs, as shown in Fig. S2. From Fig. S2A, the surface of untreated corn stover was smooth and structured, and the fibre bundles were neat, compacted and naturally stretched. As shown in Fig. S2B, the integrity of the corn stover surface was partially destroyed, forming various pits or holes on the surface of the corn stover after bio-pretreatment by *M*.*V*. These changes indicated that different degrees of degradation occurred on the surface of corn stover after bio-pretreatment due to the efficient degradation of lignocellulose caused by the enzymes secreted by *M*.*V*. during the growth process in corn stover. The results of Sun were similar, with SEM images showing the porosity on the surface of corn stover following pretreatment with *T*. *hirsute yj9*^38^. To further quantify the size of the pores on the surface of pretreated corn stover by *M*.*V*., BET Surface Area analysis was conducted, and the results are shown in Table S1. Compared to untreated corn stover, the BET Surface Area and Langmuir Surface Area of pretreated corn stover increased to 5.7728 m^2 ^g^−1^ and 10.3789 m^2^ g^−1^, respectively. No pores could be observed on the surface of untreated corn stover. The porosity of pretreated corn stover was increased, cellulase was able to contact with the cellulose directly through the porous structure and allowed the enzyme hydrolysis to be enhanced^39^. Tang confirmed that after organic amine catalytic organosolv pretreatment, the specific surface area of the n-propylamine group increased from 1.19 to 1.82 m^2^ g^−1^ compared with untreated corn stover, which resulted in smaller changes to the surface of the corn stover than did *M*.*V*. pretreatment^40^.

**Figure 6:**
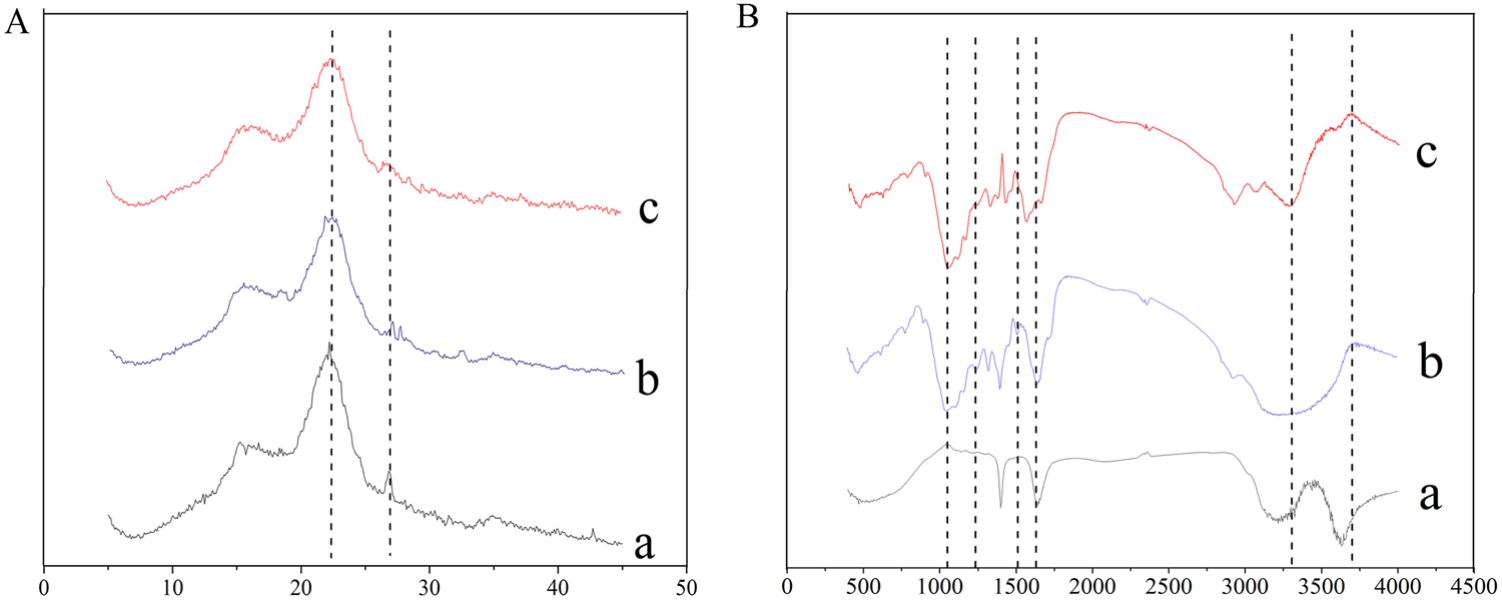
XRD and FTIR of untreated and bio-pretreated corn stover. (**A**) Stands for XRD; (**B**) stands for FTIR; a stands for untreated corn stover; b stands for bio-pretreated corn stover with *P*.*C*.; c stands for bio-pretreated corn stover with *M*.*V*.

XRD was used to determine the crystallinity of the corn stover. After bio-pretreatment, the diffraction peak intensity at 22.3° was significantly decreased, and the diffraction peak disappeared at 26.9°, indicating that the fermentation of *M*.*V*. had a great effect on the crystallinity of corn stover, and the crystallinity of lignocellulose also declined to a certain degree (as shown in Figure 6A). FTIR was used to determine the changes of chemical group of corn stover (as presented in Figure 6B). Compared to the untreated corn stover, the absorption at 1603 and 1423 cm^−1^ (aromatic skeletal vibrations) declined after bio-pretreatment, demonstrating the removal of lignin. The peaks at 1220–1240 cm^−1^ (the ether bond between lignin phenylpropane monomers) represents phenols, ethers, alcohols and esters which were decomposed from lignin. The increased intensity of these peaks indicated the increase of the lignin degradation products. Though lignin could be totally decomposed to CO_2_ and H_2_O, partial degradation products of small molecules could also be detected as reported by Singh who proved that Lac treatment resulted in an ~3 fold increase in vanillin levels together with increased levels of 4-hydroxybenzoate and 2,6 dimethoxybenzene-1,4-diol^41^. The peaks at 1030 cm^−1^ (C-O-H stretching of primary and secondary alcohols of cellulose) were enhanced by the pretreatment with *M*.*V*. and *P*.*C*., which indicated that the proportion of cellulose was increased^40^. The broad absorption band located from 3300 to 3500 cm^−1^ corresponded to the –OH stretching vibration signals and increased remarkably after pretreatment with *M*.*V*., indicating the exposure of cellulose and hemicellulose^42^. In conclusion, *M*.*V*. fermentation had a significant effect on the structure and composition of corn stover, and the lignin in corn stover was degraded partly through this bio-pretreatment process.

### Enzymatic saccharification of corn stover pretreated with *M*.*V.*

Based on previous research, the pretreatment of *M*.*V*. fermentation could efficiently deplete the lignin in corn stover because of the role of the secreted enzymes during the process of fermentation. The synergistic effect of the three enzymes (Lac, LiP, and MnP) might have produced highly efficient lignin depletion. However, the presence of Lac would inhibit cellulase activity during subsequent enzymatic hydrolysis process^36^. Therefore, in order to improve the efficiency of enzymatic hydrolysis, several steps were carried to inactivate Lac. Two reported methods, which could effectively remove mycelium and inactive Lac after bio-pretreatment, were compared in this research.

**Figure 7:**
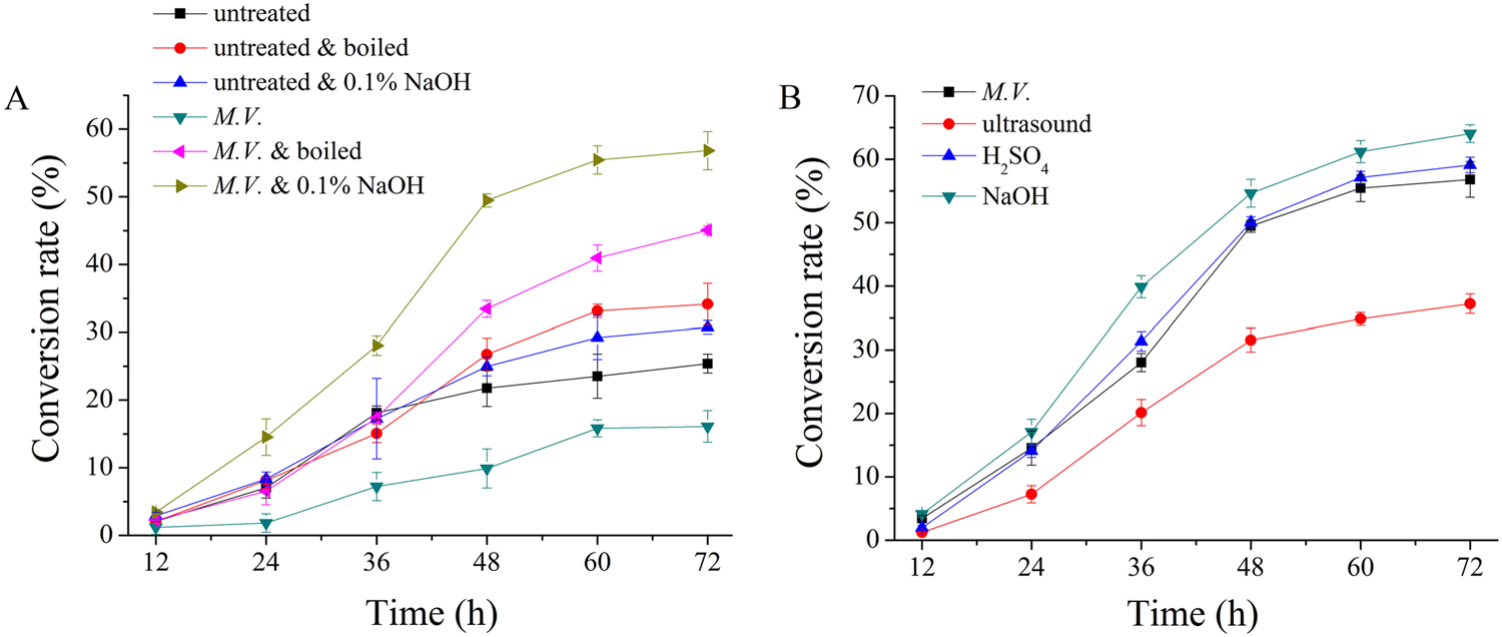
Comparison of the enzymolysis effects of pretreated corn stover by different methods during enzymatic hydrolysis for 72 h. (**A**) Stands for the enzymolysis effects of bio-pretreated corn stover with different regeneration methods; (**B**) stands for the enzymolysis effects of corn stover by different pretreatment methods. Each value is the mean of the three replicates.

The enzymatic hydrolysis of untreated corn stover, *M*.*V*. pretreated corn stover without regeneration, *M*.*V*. pretreated corn stover boiled with deionized water, *M*.*V*. pretreated corn stover washed with 0.1% NaOH solution, untreated corn stover boiled with deionized water and untreated corn stover washed with 0.1% NaOH solution were evaluated to determine whether the effect of enzymatic enhancement is derived from biological pretreatment. The results are shown in Figure 7A. The content of lignocellulose is shown in Table S2. As shown in Figure 7A, we observed that the conversion rate of untreated corn stover was only 25.38% after enzymatic hydrolysis for 72 h, while the conversion rate of bio-pretreated corn stover, which dried directly after 14 d fermentation, was 16.09%, which was 36.60% lower than the control. This might be due to the presence of lignin-degrading enzymes with the ability to inhibit the role of cellulases. This result also indicated that the removal of mycelium and the inactivation of Lac were necessary. The conversion rates of corn stover pretreated with *M*.*V*. and boiled in deionized water and pretreated with *M*.*V*. and washed in 0.1% NaOH were 45.07% and 56.81%, respectively, which were 77.58% and 123.84% higher than that of the untreated corn stover. In contrast, the conversion rates of untreated corn stover only boiled with deionized water and washed with 0.1% NaOH solution were 32.16% and 30.74%, which were only 26.71% and 21.12% higher than those of the control, much lower than those of *M*.*V*. pretreatment, indicating that the effect of enzymatic enhancement was mainly attributed to *M*.*V*. pretreatment, instead of corn stover regeneration process. Furthermore, it could also be concluded from Table S2 that lignin degradation slightly increased (2.34% and 0.65% for untreated and *M*.*V*. pretreated, respectively) after washing with NaOH solution because lignin was also soluble in NaOH. Nevertheless, the degradation of lignin was mainly due to the biological treatment according to the data. These comparisons indicated that *M*.*V*. pretreatment increased the conversion of cellulose significantly. Both boiling and washing with a weak NaOH solution would remove the inhibition of Lac and improve the efficiency of enzymatic hydrolysis, which agreed with the results of Wang, who proved that boiling after pretreatment could eliminate the repression of ligninase on cellulase activity and could thus facilitate the hydrolysis of corn stover by cellulase and xylase^43^. Zhou also mentioned that washing the fermentation blends with 0.1% NaOH was an effective method^21^. Our results also proved that the bio-pretreated corn stover washed with 0.1% NaOH solution was a better method for corn stover regeneration.

The enzymolysis effects of pretreatment with *M*.*V*., ultrasound, 1% sulphuric acid solutions, and 2% NaOH solutions were compared. The results are shown in Figure 7B. The conversion rate of cellulose to glucose after enzymatic hydrohysis was 56.81%, 37.24%, 59.12%, and 64.05% for the pretreatment of corn stover using *M*.*V*., ultrasound, 1% sulphuric acid solutions, and 2% NaOH solutions, respectively. The conversion rate of *M*.*V*. pretreatment corn stover was 52.55% higher than that of ultrasonic pretreatment corn stover and only 3.91% lower than that of sulphuric acid pretreatment corn stover. Wang reported that sugar yield of corn stover could be increased by 50.2% after a 2 d-pretreatment with crude ligninolytic enzymes from *P*.*C*. and *Coridus versicolor*^43^. Kim proved that 48.7% glucose yield was obtained for wet disk milling pretreated corn stover^44^. A two-stage dilute acid followed by wet-milling pretreatment process resulted in 64.0% glucose yield^45^. Liu and Wyman reported a glucose yield of 50.5% using hydrothermal treated corn stover at 200 °C^46^. Compare to these pretreatment methods, *M*.*V*. pretreatment achieved a high conversion rate of cellulose for corn stover with the following advantages: (1) improve the formation of sugars or the ability to subsequently form sugars by hydrolysis, (2) avoid the degradation or loss of carbohydrate, and (3) be cost-effective with less pollution, low temperature, low pressure, low energy consumption. In conclusion, our results indicated that pretreatment with *M*.*V*. was an efficient method to enhance the enzymatic hydrolysis of cellulose in corn stover.

## Conclusions

This study demonstrated the feasibility of using screened fungus *M*.*V*. pretreatment of corn stover to improve the efficiency of enzymatic hydrolysis, and proved that *M*.*V*. has high-efficiency lignin depletion capability, which secretes three lignin-degrading enzymes simultaneously during bio-pretreatment. Physical and chemical analysis on the structure and composition of corn stover showed that bio-pretreatment with *M*.*V*. depleted lignin more efficiently than that with *P*.*C*. The conversion rate of cellulose reached 56.81% after bio-pretreatment, which was 123.84% higher than that of untreated corn stover. This study investigated a strain in bio-pretreating corn stover and provided an efficient bio-pretreatment procedure.

## Materials and Methods

### High-throughput screening for lignin-degrading strains

Samples were collected from soil, rotted leaves and rotted wood of the Changbai Mountain Nature Reserve forest in the Northeast of China; a total of 29 samples were collected. Two hundred milligram samples were suspended in 1 ml of sterile saline (0.9%, w/v, NaCl). After sedimentation, 100 µl of the clear supernatant (undiluted, 10- or 100 -fold diluted in sterile saline) was spread onto Potato Dextrose Agar Medium (PDA, 200 g l^−1^ potato, 20 g l^−1^ glucose, and 18 g l^−1^ agar). The plates were incubated at 29 °C. Single colonies were isolated on PDA plates after the colonies were grown^47^. Isolates were incubated into alkali lignin culture medium (1.0 g l^−1^ alkali lignin, 2.0 g l^−1^ (NH_4_)_2_SO_4_, 2.0 g l^−1^ KH_2_PO_4_, 0.3 g l^−1^ MgSO_4_, 0.3 g l^−1^ CaCl_2_, 0.5 g l^−1^ NaCl, 0.005 g l^−1^ FeSO_4_, 0.016 g l^−1^ MnSO_4_, 0.017 g l^−1^ ZnCl_2_) at 220 rpm at 29 °C for 96 h in a 24-well deep well plate. The removal rate of alkali lignin was determined. Isolates showing strong growth in the alkali lignin medium and having the ability to remove the alkali lignin were transferred onto PDA plates, which were stored at 4 °C and used to pretreat the corn stover. Three parallel tests were run for each sample.

An alkali lignin solution with concentrations of 20, 40, 60, 80, 100 mg l^−1^ was prepared; the maximum absorption peak was determined to be 230 nm by measuring the solution with a spectrophotometric microplate reader. The standard curve is shown in Fig. S3. The content of alkali lignin in the solution was calculated by the standard curve. The removal rate of alkali lignin was as follows:

$${\rm{Removal}}\,{\rm{rate}}\,( \% )=({{\rm{C}}}_{1}-{{\rm{C}}}_{2})/{{\rm{C}}}_{1}\ast 100 \% $$

C_1_: The content of alkali lignin solution without inoculation; C_2_: The content of alkali lignin solution after the strain degradation.

### Fungal pretreatment procedure

The middle part of corn stover (Xianyu 335 species) was selected for this experiment. The corn stover was cleaned, milled, screened in a 60–80 mesh and dried to a constant weight. The process of fungal pretreatment was performed in 24-well deep well plates by Solid-state fermentation (SSF), which has been shown to produce a much higher concentration of enzymes^21^. Then, 0.5 g of corn stover and 1.5 ml of inorganic nutrient solution (2.0 g l^−1^ (NH_4_)_2_SO_4_, 2.0 g l^−1^ KH_2_PO_4_, 0.3 g l^−1^ MgSO_4_, 0.3 g l^−1^ CaCl_2_, 0.5 g l^−1^ NaCl, 0.005 g l^−1^ FeSO_4_, 0.016 g l^−1^ MnSO_4_, 0.017 g l^−1^ ZnCl_2_) were mixed and sterilized at 121 °C for 20 min. One 5 mm diameter agar disc of 7-day-old strains was inoculated into the corn stover culture. The 24-well plate was covered and cultivated at 29 °C for 14 days. Three parallel tests were run for each sample.

### Enzyme extraction and activity assay

After 14 d of SSF, 4 ml of sterile water was added to each well, and extracted at 450 rpm for 2 h before being centrifuged at 3500 rpm for 10 min. Crude extraction was used for enzymatic activity determination. The enzymatic activities of Lac, LiP and MnP were determined according to the procedures of Chairattanamanokorn, Tien and Warrishi^48–50^. The Lac, Lip and MnP activity was measured using 2,2′-azino-bia (ethylbenzthiazoline-6- sulphonic acid) (ABTS), veratryl and alcohol as substrates. One unit of enzyme activity was defined as the amount of enzyme that transformed 1 μmol of substrate in one minute. The activity of each enzyme was expressed in units of U g^−1^ dry biomass of corn stover^29^. Three parallel tests were run for each sample.

### Corn stover regeneration

Hot water boiling and weak alkali washing was used for pretreated corn stover regeneration (recovery of the polysaccharides as solids), including inactivated the Lac produced by the strain and removed the mycelium during the bio-pretreatment process. For the first method, the fermentation blends were boiled with deionized water for 15 min and the pretreated corn stover was washed and dried to a constant weight^43^. For the second method, the fermentation blends were washed with 0.1% NaOH solution for 1 h, and the pretreated corn stover was washed to a neutral pH^21^. Untreated corn stover was also treated by both methods. For the evaluation of 64 lignin-depleting fungi in 24-well plates, only washed by 0.1% NaOH solution was conducted, and for the pretreatment with *M*.*V*., both of these two methods were compared.

### Comparison with different pretreatment methods

Ultrasound^51^, 1% sulfuric acid (H_2_SO_4_) solutions^52^, and 2% NaOH solutions^53^ were adopted to pretreat corn stover according to the conditions of reported protocol for each method. After pretreatment, corn stover was washed to neutral pH with deionized water and dried to constant weight for subsequent enzymatic hydrolysis.

### Enzymatic saccharification assay

The conversion of cellulose to glucose was conducted to estimate the efficiency of the bio-pretreatment. Hydrolysis of corn stover was conducted with 3% (w/w) cellulose in corn stover and 0.5% (v/v) 50 mM citrate buffer (pH 4.8)^21,54^. The mixture was sterilized at 121 °C for 20 min. The suspension was further supplemented with 30 fpu g^−1^ cellulose from commercial cellulases (Novozymes) and 60 U g^−1^ β-glucosidase (Novozymes SP188). The 24-well was incubated at 50 °C and 160 rpm in water bath shaker for 72 h. The released glucose in the supernatants was determined using the Glucose RTU kit (Shanghai Rongsheng Biotech Co., Ltd.) to calculate the conversion rate of cellulose to glucose. Three parallel tests were run for each sample.

### DNA extraction, ITS sequencing and phylogenetic classification

The genomic DNA of the strain was isolated using the NucleoSpin ® Tissue Kit according to the manufacturer’s instructions for fungus. The ITS sequences were PCR amplified. Nearly complete ITS sequences (556) were analysed with the BLASTN program (National Center for Biotechnology Information; http://www.ncbi.nlm.nih.gov) to phylogenetically assign the isolates.

### Large-scale fermentation of corn stover by fungi

10 g of corn stover and inorganic nutrient solution (2.0 g l^−1^ (NH_4_)_2_SO_4_, 2.0 g l^−1^ KH_2_PO_4_, 0.3 g l^−1^ MgSO_4_, 0.3 g l^−1^ CaCl_2_, 0.5 g l^−1^ NaCl, 0.005 g l^−1^ FeSO_4_, 0.016 g l^−1^ MnSO_4_, 0.017 g l^−1^ ZnCl_2_) were mixed at the ratio of 1:3(w/v), and sterilized at 121 °C for 20 min. Strains were inoculated into the corn stover culture with 2% inoculation amount. Then the mixture cultivated at 29 °C for 14 days. Three parallel tests were run for each sample.

### Composition analysis of the corn stover

The cellulose, hemicellulose and lignin content of untreated and bio-pretreated corn stover was analysed based on a method described by the National Renewable Energy Laboratory (NREL, 2008). After two steps of acid hydrolysis, the released glucose and xylose were measured using the Glucose RTU kit and D-xylose kit. Then, the content of cellulose and hemicelluloses was calculated. Acid-insoluble lignin and acid-soluble lignin were also measured. The total phenolic content of the supernatants of corn stover before and after bio-pretreatment was determined according to the Folin-Ciocalteu method^55^. Twenty microlitres of the sample and the serial standard solution were diluted with 88 μl of water in a 96-well microplate. The plate was incubated for 5 min at room temperature in dark conditions after the addition of 12 μl of Folin-Ciocalteu reagent. The reaction was stopped with 80 μl of 7.5% (w/v) sodium carbonate solution, and the plate was incubated for 2 h at room temperature in the dark. The absorbance was measured at 765 nm with a spectrophotometric microplate reader. Three parallel tests were run for each sample.

### Effects of bio-pretreatment on the structure of the corn stover

The untreated and bio-pretreated corn stover was dehydrated using a freeze dryer. The different surface morphologies of the untreated and bio-pretreated corn stover were characterized using a scanning electron microscope (SEM). The porosity and specific surface area of the untreated and bio-pretreated corn stover were determined using a Brunauer-Emmet-Teller (BET) surface area analyser. An X-ray diffractometer (XRD) and Fourier transform infrared (FTIR) spectroscopy analysis were conducted to evaluate changes in the crystallinity and functional groups of corn stover after bio-pretreatment^56^. Three parallel tests were run for each sample.

## Electronic supplementary material

Supplementary Information
