# Supplementary material for: Evaluation of Screened Lignin-degrading Fungi for the Biological Pretreatment of Corn Stover
Source: Sci Rep. 2018 Mar 29;8:5385. doi: 10.1038/s41598-018-23626-6 (PMC5876370; doi:10.1038/s41598-018-23626-6)
Supplement: Supplementary file 1 — Supplementary Information [file 41598_2018_23626_MOESM1_ESM.pdf]

Supplementary Information for Evaluation of Screened Lignin-degrading Fungi for the Biological Pretreatment of Corn Stover

Yingjie Su, Xiaoxiao Yu, Yang Sun, Gang Wang, Huan Chen, Guang Chen*[1]

1. Growth condition of *Myrotheium verrucaria*

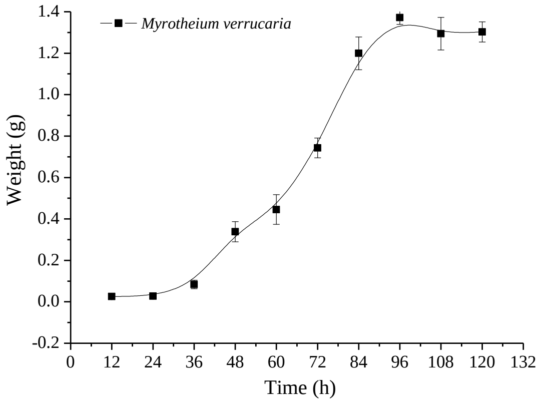

Fig. S1 Growth curve of *Myrotheium verrucaria*

2. Determination of the structure of pretreated corn stover

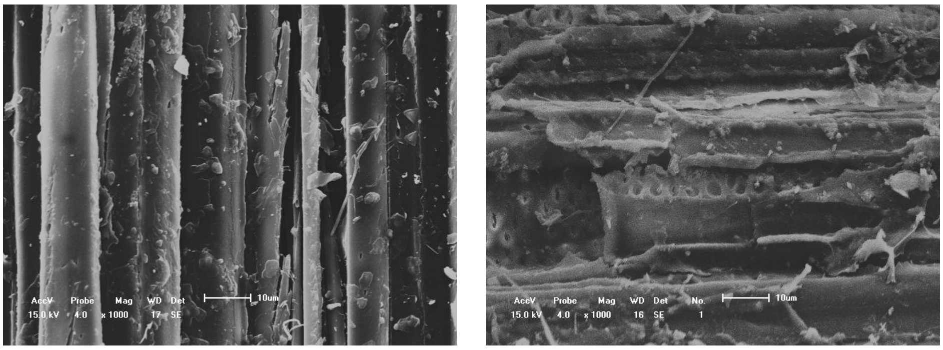

A B

Fig. S2 Scanning electron microscopy (SEM) of untreated and pretreated corn stover. A stands for the surface of untreated corn stover; B stands for the surface of bio-pretreated corn stover by *Myrotheium verrucaria.*

[1] College of Life Sciences, Jilin Agricultural University, Changchun, 130118, China. Correspondence and requests for materials should be address to G.C. (email: chg61@163.com)

3. Nitrogen adsorption isotherms for corn stover

Table S1 Nitrogen adsorption isotherms for the bio-pretreated corn stover with *Myrotheium verrucaria at* 77K.

Summary Report	
Surface area	
BET Surface area	5.7728 m^2/g
Langmuir Surface Area	10.3789 m^2/g
BJH Adsorption cumulative surface area of pores between 1.7000 nm and 300.0000 nm diameter	1.369 m^2/g
Pore volume	
BJH Adsorption cumulative volume of pores between 1.7000 nm and 300.0000 nm diameter	0.003527 cm^3/g
Pore size	
Adsorption average pore width (4V/A by BET)	3.65173 nm
BJH Adsorption average pore diameter (4V/A)	10.3073 nm

4. Composition analysis of the corn stover

Table S2. Lignocellulose content of different corn stover

Sample	Cellulose (%)	Hemicellulose (%)	Lignin (%)
untreated	38.34±1.05	18.95±0.67	22.57±2.13
Untreated & boiled	40.99±1.21	19.46±1.08	23.17±0.21
Untreated & washed	41.12±0.35	18.21±0.63	20.23±1.70
M.V. pretreated	48.79±0.38	24.60±2.79	16.99±0.49
M.V. pretreated & boiled	45.84±0.98	24.83±0.89	17.71±0.49
M.V. pretreated & washed	53.59±2.07	21.92±1.38	16.34±1.12

5. Determination of alkali lignin content

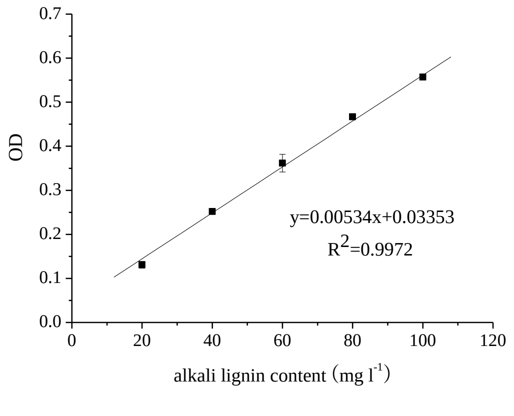

Fig. S3 Standard curve of alkaline lignin.
